# Broadly Protective Adenovirus-Based Multivalent Vaccines against Highly Pathogenic Avian Influenza Viruses for Pandemic Preparedness

**DOI:** 10.1371/journal.pone.0062496

**Published:** 2013-04-30

**Authors:** Sai V. Vemula, Yadvinder S. Ahi, Anne-Marie Swaim, Jacqueline M. Katz, Ruben Donis, Suryaprakash Sambhara, Suresh K. Mittal

**Affiliations:** 1 Department of Comparative Pathobiology, College of Veterinary Medicine, Purdue University, West Lafayette, Indiana, United States of America; 2 Bindley Bioscience Center, Purdue University, West Lafayette, Indiana, United States of America; 3 Influenza Division, Centers for Disease Control and Prevention, Atlanta, Georgia, United States of America; College of Medicine, Hallym University, Republic of Korea

## Abstract

Recurrent outbreaks of H5, H7 and H9 avian influenza viruses in domestic poultry accompanied by their occasional transmission to humans have highlighted the public health threat posed by these viruses. Newer vaccine approaches for pandemic preparedness against these viruses are needed, given the limitations of vaccines currently approved for H5N1 viruses in terms of their production timelines and the ability to induce protective immune responses in the absence of adjuvants. In this study, we evaluated the feasibility of an adenovirus (AdV)-based multivalent vaccine approach for pandemic preparedness against H5, H7 and H9 avian influenza viruses in a mouse model. Replication-defective AdV vectors expressing hemagglutinin (HA) from different subtypes and nucleoprotein (NP) from one subtype induced high levels of humoral and cellular immune responses and conferred protection against virus replication following challenge with H5, H7 and H9 avian influenza virus subtypes. Inclusion of HA from the 2009 H1N1 pandemic virus in the vaccine formulation further broadened the vaccine coverage. Significantly high levels of HA stalk-specific antibodies were observed following immunization with the multivalent vaccine. Inclusion of NP into the multivalent HA vaccine formulation resulted in the induction of CD8 T cell responses. These results suggest that a multivalent vaccine strategy may provide reasonable protection in the event of a pandemic caused by H5, H7, or H9 avian influenza virus before a strain-matched vaccine can be produced.

## Introduction

Occasional reports of human infection with both low and highly pathogenic avian influenza A viruses of H5, H7, and H9 subtypes underscore the public health threat and pandemic potential posed by these avian influenza subtypes [1], [2]. Since their emergence in Asia over a decade ago, highly pathogenic avian influenza H5N1 viruses have spread to over sixty countries on three continents and are endemic among poultry in South East Asia and Africa. Additionally, both low and highly pathogenic H7 viruses continue to cause sporadic outbreaks, whereas H9 viruses are endemic among poultry globally [3]. As of February 2013, more than 700 cases of human infection with the avian influenza viruses from H5, H7, and H9 subtypes have been reported to the World Health Organization (WHO) from fifteen countries. Of these, infections with H5N1 viruses account for up to 85% of the cases with a case fatality rate of approximately 60% [4]. Although human-to-human transmission has been infrequent and limited, genetic assortment between any of these avian viruses and a circulating human influenza virus and/or acquisition of key mutations in the hemagglutinin (HA) that confer binding to human-like sialic acid receptors and other genes could result in the generation of a novel pandemic influenza virus with the capacity to infect and effectively transmit among humans with little or no immunity to the new virus [5], [6]. The influenza viruses responsible for the pandemics of 1918, 1958 and 1967 were all thought to have originated either through genetic assortment between avian and human viruses or direct transmission of an avian virus to humans [7]. The recent 2009 H1N1 pandemic influenza virus possesses genes derived from avian, human and swine influenza viruses through multiple reassortment events [8].

Vaccination remains the most effective and economically prudent strategy to combat the threat posed by avian influenza viruses with pandemic potential. For pandemic preparedness purposes, vaccines against H7, H9 and multiple clades of H5N1 viruses have been developed and clinically evaluated, and in the case of H5N1 vaccines, stockpiled by national and international human health agencies. The use of an adjuvant is necessary to heighten and broaden neutralizing antibody responses elicited by H5N1 inactivated split or subunit vaccines to achieve cross-reactivity across H5N1 clades and subclades [9], [10]. Similar concerns exist with vaccines developed against H7 and H9 virus subtypes. Recently, a cell culture-derived H7N1 vaccine was found be to poorly immunogenic in humans in the absence of a suitable adjuvant [11]. Furthermore, low cross-reactivity against heterologous viruses from other clades despite inclusion of MF59 adjuvant has been reported in a few clinical trials with H9N2 vaccines [12]. Hence, newer vaccine approaches with the potential to induce both humoral and cellular immune responses are needed that confer protection against a broad range of influenza viruses emerging from animal reservoirs.

In the present study, the feasibility of an adenovirus (AdV) -based multivalent vaccination approach against multiple avian influenza viruses of the H5, H7, and H9 subtypes was evaluated in a mouse model. Replication-incompetent AdV vectors expressing HA derived from H5, H7, and H9 avian influenza subtypes were generated and evaluated for their ability to induce protective immunity against heterologous viruses when used alone or in combination as a multivalent vaccine formulation. The influenza virus nucleoprotein (NP) has been shown to be a target of virus-specific CD8+ T cells that contribute to virus clearance following infection [13]. Since NP is relatively conserved, the cell-mediated immune (CMI) response generated against NP is usually broad and cross-protective across influenza A virus subtypes [14], [15]. Therefore, to further enhance the cross-protective efficacy of the multivalent vaccines, an AdV-vector expressing NP of a H5N1 virus was included into the vaccine formulation. A multivalent HA vaccine containing HA of H5, H7 and H9 avian influenza viruses induced effective protection against challenge with antigenically distinct influenza A viruses from all the three avian subtypes. Furthermore, inclusion of NP into the multivalent HA vaccine formulation induced cross-protection against heterosubtypic virus challenge, in addition to homosubtypic viruses. Our findings highlight the utility of AdV vector-based vaccines to generate multivalent vaccine formulations with broad cross-protection against multiple avian influenza virus subtypes that have the potential to cause a pandemic.

## Materials and Methods

### Ethics Statement

The Purdue University Biosafety Committee and Animal Care and Use Committee approved the protocol for all animal studies described in this manuscript and conducted at Purdue University, under the auspices of the Institutional Animal Care and Use Committee (IACUC) #A3231-01 which is supported by the American Association for Laboratory Animal Science (AALAS). All animals were humanely euthanized by an overdose of anesthetic (ketamine/xylazine). The 293 and MDCK cell lines were obtained commercially from American Type Culture Collection (ATCC), and the 293Cre cell line was obtained from Merck & Co., Inc., Whitehouse Station, NJ. The BHH2C cell line which was created in the PI’s laboratory using a combination of commercially available cell lines, MDBK and 293. The use of all human cell lines and the construction of BHH2C hybrid cell line were with permission from the Purdue University Institutional Review Boards (IRB) formed in accordance with federal regulations. A Research Exemption was obtained for the use of commercially available human cell lines. The IRBs are a unit of the Human Research Protection Program (HRPP) which is housed within the Office of Research Administration (ORA).

### Cells and Viruses

MDCK (Madin-Darby canine kidney), 293 [human embryonic kidney cells expressing human AdV serotype 5 (HAdV5) E1 gene products], 293Cre [293 cell line that constitutively expresses Cre recombinase (a gift from Merck & Co., Inc.)], and BHH2C (bovine-human hybrid clone 2C) [16] cells were grown as monolayer cultures in minimum essential medium (MEM) (Life Technologies, Gaithersburg, MD) supplemented with 10% fetal bovine serum (Hyclone, Logan, UT) and gentamycin(50 µg/ml). The following influenza A viruses that are generated by reverse genetics (RG) technology were used in this study: A/Egypt/3300-NAMRU3/2008 (H5N1)-PR8-IDCDC-RG13 [Egypt/08]; A/Vietnam/1203/2004 (H5N1)-PR8/CDC-RG [VN/1203]; A/chicken/Vietnam/NCVD-016/2008(H5N1)-PR8-IDCDC-RG12 [VN/08]; A/turkey/Virginia/2002 (H7N2)-PR8-IBCDC-5 [TK/VA][17]; A/chicken/Hong Kong/G9/1997 (H9N2)-PR8-IBCDC-2 [G9/97]; and A/Aichi/2/1968 (H3N2)-PR8 [X-31]. The 2009 H1N1 pandemic influenza virus and the H9N2 influenza virus strain used in this study were: A/California/08/2009 (H1N1) [pH1N1], and A/Hong Kong/1073/1999 (H9N2) [G1/99], respectively.

### Generation of AdV Vector-based Vaccine Constructs

HAdV vector-based monovalent vaccine constructs, HAd-H1HA (H1HA), HAd-H7HA (H7HA), and HAd-H9HA (H9HA), were generated after placing the whole length coding region of the HA gene of H1, H7, or H9 subtype influenza virus, A/California/04/2009 (H1N1), A/Netherlands/219/2003 (H7N7), or A/chicken/Hong Kong/G9/1997(H9N2), respectively. The gene was under the control of the human cytomegalovirus (HCMV) immediate early promoter and the bovine growth hormone (BGH) polyadenylation signal (PolyA) and was inserted in the early (E) 1 region of HAdV5 genome (containing deletions in E1 and E3 regions) using a Cre-Lox recombination system in a 293Cre cell line [18]. Similarly, a bivalent vaccine construct, HAd-1203HA-05HA (H5b), was generated after inserting the full-length coding region of the HA gene of clade 2 A/Indonesia/05/2005 (H5N1) virus under the control of the HCMV promoter and the BGH PolyA along with the full-length coding region of the HA gene of clade 1 A/Vietnam/1203/2004 (H5N1) virus under the control of the murine CMV (MCMV) promoter and the simian virus 40 (SV40) polyA in the E1 region. Similarly, another bivalent vaccine construct, HAd-H7HA-H9HA (H7H9b), was generated containing the full-length coding regions of HA from the A/Netherlands/219/2003 (H7N7) and the A/Chicken/Hong Kong/G9/1997 (H9N2) viruses, respectively. The polybasic cleavage sites present in the HA genes of A/Netherlands/219/2003 (H7N7), A/Indonesia/05/2005 (H5N1) and A/Vietnam/1203/2004 (H5N1) viruses was modified to match the cleavage sites of low*-*pathogenic strains. All vaccine constructs were purified by cesium chloride density-gradient centrifugation and titrated by plaque assays on the BHH2C cell line as described [16]. The construction and characterization of HAd-1203HA (1203HA), HAd-05HA (05HA), and HAd-NP (NP) has been previously described [19]. The AdV vector particle count (VP) was determined spectrophotometrically by measuring absorbance at 260nm ([16], [20]. The plaque-forming unit (PFU) to VP ratio for vectors used in this study was in the range of 75–100 (data not shown).

### Animal Inoculation and Protection Studies

All animal studies were conducted following guidelines and approval from Institutional Biosafety Committee and Institutional Animal Care and Use Committee at Purdue University. Experiments involving influenza viruses were conducted under Biosafety Level 2+ containment. To assess the breadth of immune response, groups of six-to-eight week-old female BALB/c mice (7+25 animals/group) obtained from Harlan Sprague Dawley Inc., Indianapolis were inoculated intramuscularly (i.m.) with 10^8^ plaque-forming units (PFU) of each of mono or bivalent AdV vector alone: H1HA; 1203HA; 05HA; H7HA; H9HA; H5b; H7H9b or used in these combinations: H5b+H7H9b; H5b+NP; H7H9b+NP; H5b+H7H9b+NP; and H5b+H7H9b+H1HA (combination formulation contained 10^8^ PFU of each AdV vector) twice at four-week intervals. In this study we used a 10-fold higher vaccine dose than previously used to induce protective immune responses in mice [21]. Control groups received the same dose of the empty vector, HAd-ΔE1E3 (ΔE1E3). Four weeks after the final immunization, blood samples were collected by retro-orbital puncture to evaluate the development of HA-specific humoral responses. Seven animals from each group were euthanized by an overdose of anesthetic (ketamine/xylazine), and the spleens were collected to evaluate the induction of HA- and NP-specific cell-mediated immune responses. Five animals from each group were challenged intranasally (i.n.) with 100 50% mouse infectious doses (MID_50_) of influenza viruses, Egypt/08, TK/VA [17], G1/99 [22], pH1N1 or X-31. Three days post-challenge, the mice were euthanized, and the lungs were collected to determine viral titers to evaluate protective efficacy**.** Briefly, thawed lung tissues were homogenized in 1 ml of sterile phosphate-buffered saline (PBS), and then 10-fold serially diluted lung homogenates were used to infect MDCK cells seeded in 96-well plates. After incubation for 72 hours (h) at 37°C, the HA activity of the culture supernatants was determined by hemagglutination of turkey red blood cells (TRBC). The limit of virus detection was 0.5 log_10_ 50% tissue culture infectious dose (TCID_50_) per ml.

### Hemagglutination Inhibition Assay

Sera from all mice were treated with a receptor-destroying enzyme (RDE) from *Vibrio cholerae* (Denka Seiken, Tokyo, Japan) at 37°C for 16 h and later heat inactivated at 56°C for 30 minutes. The presence of hemagglutination inhibition (HI) antibody was determined using four hemagglutination units of each of influenza viruses and 0.5% TRBC. The HI titer was defined as the reciprocal of the highest dilution of serum which completely prevented the agglutination of TRBC.

### Microneutralization Assay

The microneutralization assay was performed using MDCK cells and 100 TCID_50_ of each influenza virus. Serial two-fold dilutions of RDE-treated and heat-inactivated serum samples were mixed with 100 TCID_50_ of an influenza virus and incubated at room temperature for 1 h. The virus antibody mixture was then added to the monolayer of MDCK cells, and the plates were incubated for 72 h at 37°C. After incubation, the HA activity of the supernatant was assessed by HA assay with 0.5% TRBC. The virus neutralization titer was defined as the reciprocal of the highest dilution of serum which showed complete absence of TRBC agglutination. The assay was done in triplicate. We did not check the induction of antibodies by vaccine constructs against the A/Indonesia/5/2005 (H5N1)-PR8/CDC-RG virus strain as it was not available for distribution by the CDC at the request of the Government of Indonesia.

### Enzyme-linked Immunosorbent Assay (ELISA)

The protocol was similar as described previously [23]. The two peptides, P1:RIQDLEKYVEDTKIDLWSYNAELLVALENQHTIDL and P2:GVTNKVNSIIDK biotinylated at the N-terminus were commercially synthesized (CHI Scientific, Inc., Maynard, MA). Peptide P1 contains a linear epitope, recognized by the monoclonal antibody 12D1, present in the highly conserved HA2 stalk region identified by analysis of 12D1 binding to HA truncation mutants [24]. Peptide P2 contains a major portion of the complex epitope, recognized by monoclonal antibody CR6261, present with in the long-alpha helix portion of the HA molecule originally identified by structural analysis of the HA binding sites of CR6261 [25].

Streptavidin-coated 96-well plates (eBioscience, San Diego, CA) were incubated with 2 μg/ml of either P1 or P2 peptide in PBS overnight at 4°C. After blocking with PBS containing 1% bovine serum albumin (BSA), two-fold serially diluted serum samples (starting at 1:50 dilution) were added and incubated at room temperature for 3 h. The horseradish peroxidase-conjugated goat anti*-*mouse*–*IgG (Sigma-Aldrich, Inc., St. Louis, MO) at a dilution of 1:2000 was added to the wells followed by 1 h incubation at room temperature. The plates were developed with a TMB substrate (Biolegend, San Diego, CA). Optical density measurements were taken at 405 nm using an EMax microplate reader (Molecular Devices, Sunnyvale, CA) after stopping the reaction with 1 M sulfuric acid. The cut-off value was set as the mean plus 3× standard deviation of the negative control.

### ELISpot Assay

96-well flat-bottom polyvinyl chloride micro-titer plates (Millipore, Billerica, MA) were coated overnight at 4°C with an anti-mouse IFN-γ antibody (BD Bioscience, San Jose, CA). Splenocytes (5×10^5^ or 1×10^6^ cells/well) isolated from inoculated mice were cultured in the presence of HA518 (IYSTVASSL) or NP147 (TYQRTRALV) peptides (H-2Kd-restricted epitopes from influenza HA or NP, respectively) in RPMI medium (Gibco, Grand Island, NY), supplemented with 10% reconstituted fetal bovine serum for 60 h and developed according to an ELISpot protocol [26]. For mice receiving H7HA vaccines, splenocytes were stimulated with 5 μg/ml of recombinant H7 HA protein (Immune Technology, New York, NY). Splenocytes cultured in the presence of phorbol myristate acetate (PMA) and ionomycin (Sigma-Aldrich, Inc.) served as positive controls.

### HA- or NP-specific Pentamer Staining

Splenocytes isolated from mice were stained with murine MHC encoded allele Kd-specific pentamer for HA518 or NP147 epitope conjugated with phycoerythrin (PE) (Proimmune Inc., Bradenton, FL) and an anti-CD8 antibody conjugated with allophycocyanin (APC) (BD Pharmingen, San Jose, CA) as described previously [27]. B cells were stained with an anti-CD19 antibody conjugated with fluoro-isothiocyanin (FITC) and gated out in analysis. Flow cytometric analyses were done using the BD FACSCanto II (BD Bioscience, San Jose, CA) and FACSDiva software to measure the percentage of HA518 or NP147 epitope-specific CD8+ T cells among the total splenic CD8+ T cells.

### Statistical Analysis

The Kruskall-Wallis test was used for calculation of significance. The significance was set at *P*<0.05.

## Results

### Generation of AdV Vectors Expressing HA and/or NP of Influenza Viruses

**Figure 1:**
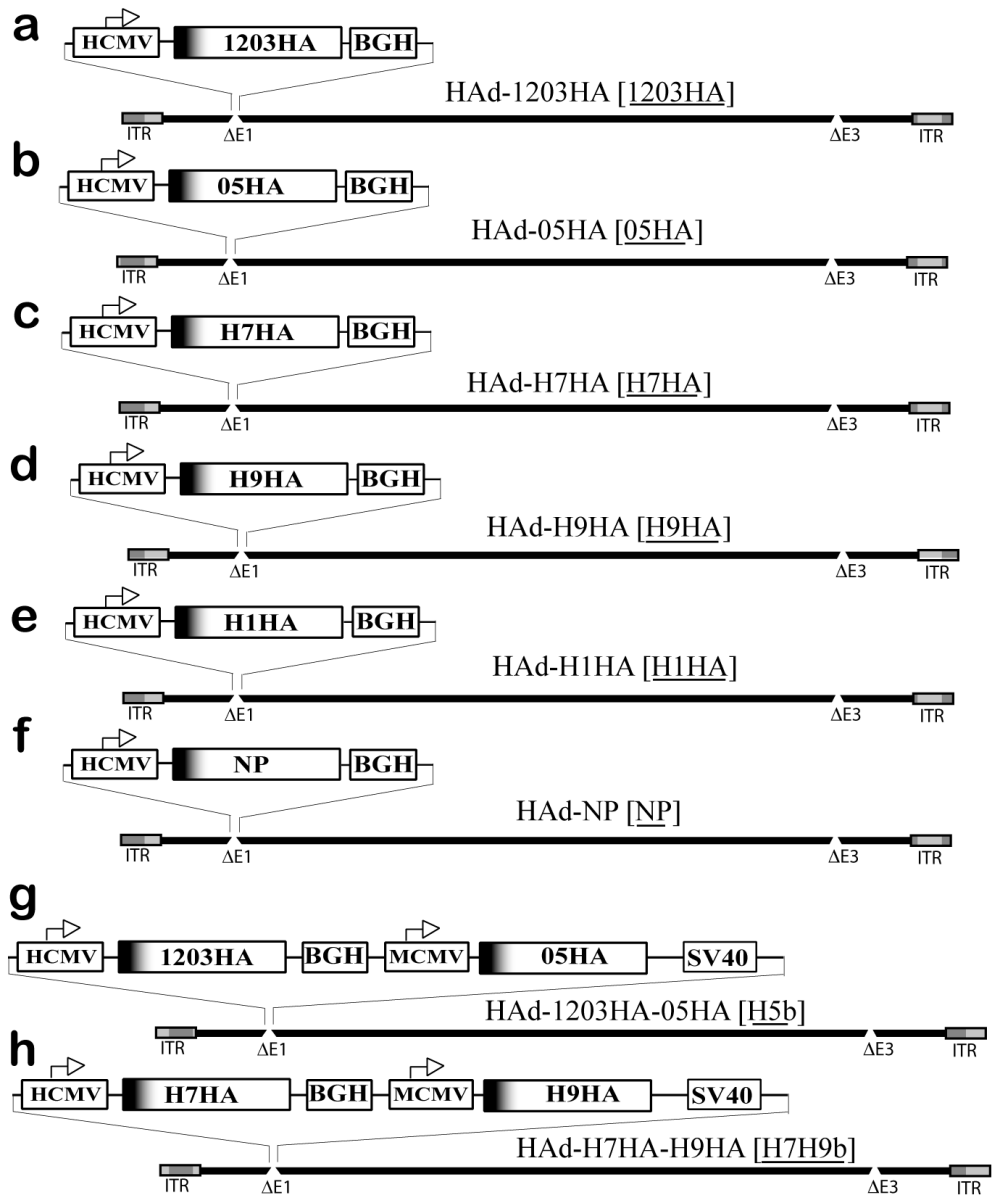
Diagrammatic representation of adenovirus vector constructs used in the study. (a). HAd-1203HA [1203HA] human adenovirus serotype 5 (HAdV5) vector containing the hemagglutinin (HA) gene of clade 1 H5N1 influenza virus (A/Vietnam/1203/2004); (b). HAd-05HA [05HA], HAdV5 vector containing the HA gene of clade 2 H5N1 influenza virus (A/Indonesia/05/2005); (c). HAd-H7HA [H7HA], HAdV5 vector containing the HA gene of A/Netherlands/219/2003 (H7N7) virus; (d). HAd-H9HA [H9HA], HAdV5 vector containing the HA gene of A/chicken/Hong Kong/G9/1997 (H9N2) virus; (e). HAd-H1HA [H1HA], HAdV5 vector containing the HA gene of A/California/04/2009 (H1N1) virus; (f). HAd-NP [NP], HAdV5 vector containing the nucleoprotein (NP) gene of clade 1 H5N1 influenza virus (A/Vietnam/1203/2004); (g). HAd-1203HA-05HA [H5b], HAdV5 vector containing the HA genes of clade 1 and clade 2 H5N1 influenza viruses; and (h). HAd-H7H9b [H7H9b], HAdV5 vector containing the HA genes of A/Netherlands/219/2003 (H7N7) and A/chicken/Hong Kong/G9/1997 (H9N2) viruses. HCMV, human cytomegalovirus immediate early promoter; MCMV, mouse cytomegalovirus immediate early promoter; BGH, bovine growth hormone polyadenylation signal; SV40, simian virus 40 polyadenylation signal; E1, early region1; E3; early region 3; and ITR, inverted terminal repeat. The vector short name used in the text, figures, and tables is underlined in the brackets.

A *Cre* recombinase–mediated site-specific recombination technique was used to insert the full-length coding regions of the HA genes of the A/California/04/2009 (H1N1), A/Netherlands/219/2003 (H7N7), or A/Chicken/Hong Kong/G9/1997(H9N2) viruses into the E1 region of the HAdV genome under the control of the HCMV promoter and the BGH PolyA to create HAd-H1HA (H1HA), HAd-H7HA (H7HA) and HAd-H9HA (H9HA) monovalent vaccine constructs, respectively (Fig. 1). The bivalent vaccine constructs, HAd-1203HA-05HA (H5b) and HAd-H7HA-H9HA (H7H9b) were generated after inserting the full-length coding regions of the HA genes of A/Vietnam/1203/2004 and A/Indonesia/05/2005 H5 subtype viruses, or A/Netherlands/219/2003 and A/Chicken/Hong Kong/G9/1997 strains of H7 and H9 viruses under the control of two separate promoters (HCMV and MCMV) and PolyA (BGH and SV40) into the E1 region of the HAdV genome. The recombinant vectors showed visible cytopathic effect (c.p.e.) five to seven days following transfection in 293cre cells. Expression of various HA in vector-infected 293 cells was confirmed by Western blotting using ferret antiserum raised against H1N1, H5N1, H7N7, or H9N2 influenza viruses respectively (data not shown). The construction and characterization of HAd-1203HA (1203HA), HAd-05HA (05HA), and HAd-NP (NP) have been described previously [19].

### Induction of Humoral Immune Responses Following Immunization with AdV Vectors Expressing HA of H5, H7, or/and H9 Avian Influenza Viruses

Serum samples collected from immunized mice were assayed for the presence of HA-specific antibodies by microneutralization and HI assays. As shown in Table 1, mouse groups that were vaccinated with the monovalent HA constructs 1203HA, H7HA, or H9HA developed geometric mean virus neutralization antibody titers of 348, 706, 1810, and 107, respectively against VN/1203, TK/VA, G9/97, or G1/99 influenza virus. Noticeable cross-reactivity was not observed with heterosubtypic viruses. In addition, vaccination with either 1203HA or 05HA failed to induce virus neutralizing antibody titers above background against the antigenically distinct H5N1 influenza viruses, Egypt/08 and VN/08 belonging to clades 2.2.1 and 7, respectively. However, mice vaccinated with a bivalent H5b construct developed antibodies against both homologous (VN/1203) and antigenically distinct (Egypt/08 and VN/08) H5N1 viruses. Further studies are needed to evaluate if co-expression of HAs from different H5N1 clades will result in a complex hetrotrimerization of HA exposing cross-protective epitopes. Vaccination with the bivalent H7H9b vaccine induced antibodies against a heterologous American lineage H7 virus (TK/VA). We did not test antibody responses against a homologous H7 virus due to the lack of availability. Moreover, H7H9b vaccination also induced substantial level of antibodies against both homologous (G9/97) and heterologous (G/99) H9N2 viruses. Although the antibody titers in the bivalent groups were two-fold lower compared to the monovalent groups, the differences were not statistically significant. The group that received the vaccine containing H5b+ H7H9b induced antibodies against viruses from all three avian influenza subtypes. In general, the antibody titers in the multivalent group were slightly lower compared to the monovalent or bivalent groups. Similar results were obtained when the serum samples from immunized mice were tested for HI titers (Table 1).

**Table 1:** Virus neutralization and hemagglutination inhibition antibody titers in mice vaccinated with AdV vector-based vaccines.

Vaccine group	VN/1203 (H5N1)		Egypt/08 (H5N1)		VN/08 (H5N1)		TK/VA (H7N2)				G9/97 (H9N2)		G1/99 (H9N2)	
	HI	VN	HI	VN	HI	VN	HI			VN	HI	VN	HI	VN
**ΔE1E3**	≤10	≤10	≤10	≤10	≤10	≤10	≤10			≤10	≤10	≤10	≤10	≤10
**1203HA**	274	348	≤10	≤10	≤10	≤10	≤10			≤10	≤10	≤10	≤10	≤10
**05HA**	≤10	≤10	≤10	≤10	≤10	≤10	≤10			≤10	≤10	≤10	≤10	≤10
**H5b**	194	302	67	107	40	35	≤10			≤10	≤10	≤10	≤10	≤10
**H7HA**	≤10	≤10	≤10	≤10	≤10	≤10	390			706	≤10	≤10	≤10	≤10
**H9HA**	≤10	≤10	≤10	≤10	≤10	≤10	≤10			≤10	1159	1810	65	107
**H7H9b**	≤10	≤10	≤10	≤10	≤10	≤10	262		422		951	1529	48	99
**H5b+H7H9b**	148	211	44	52	30	32	144	160			861	905	52	97

Mice (7 animals/group) were immunized with 1203HA, 05HA, H5b, H7HA, H9HA, H7H9b, H5b+H7H9b, or ΔE1E3 twice at four-week intervals. Four weeks post-booster inoculation, serum samples were obtained for determining virus neutralizing (VN) and hemagglutination inhibition (HI) antibody titers. The data are shown as the geometric mean titers. 1203HA, HAd-1203HA; 05HA, HAd-05HA; H5b, HAd-1203HA-05HA; H7HA, HAd-H7HA; H9HA, HAd-H9HA; H7H9b, HAd-H7HA-H9HA; H5b+H7H9b; HAd-1203HA-05HA+HAd-H7HA-H9HA; ΔE1E3, HAd-ΔE1E3; VN/1203, A/Vietnam/1203/2004 (H5N1)-PR8/CDC-RG; VN/08, A/chicken/Vietnam/NCVD-016/2008(H5N1)-PR8-IDCDC-RG12; Egypt/08, A/Egypt/3300-NAMRU3/2008 (H5N1)-PR8-IDCDC-RG13; TK/VA, A/turkey/Virginia/2002 (H7N2)-PR8-IBCDC-5; G9/97, A/chicken/Hong Kong/G9/1997 (H9N2)-PR8-IBCDC-2; G1/99, A/Hong Kong/1073/1999 (H9N2).

### Induction of Cellular Immune Responses Following Immunization with AdV Vectors Expressing HA of H5, H7 and/or H9 Avian Influenza Viruses

**Figure 2:**
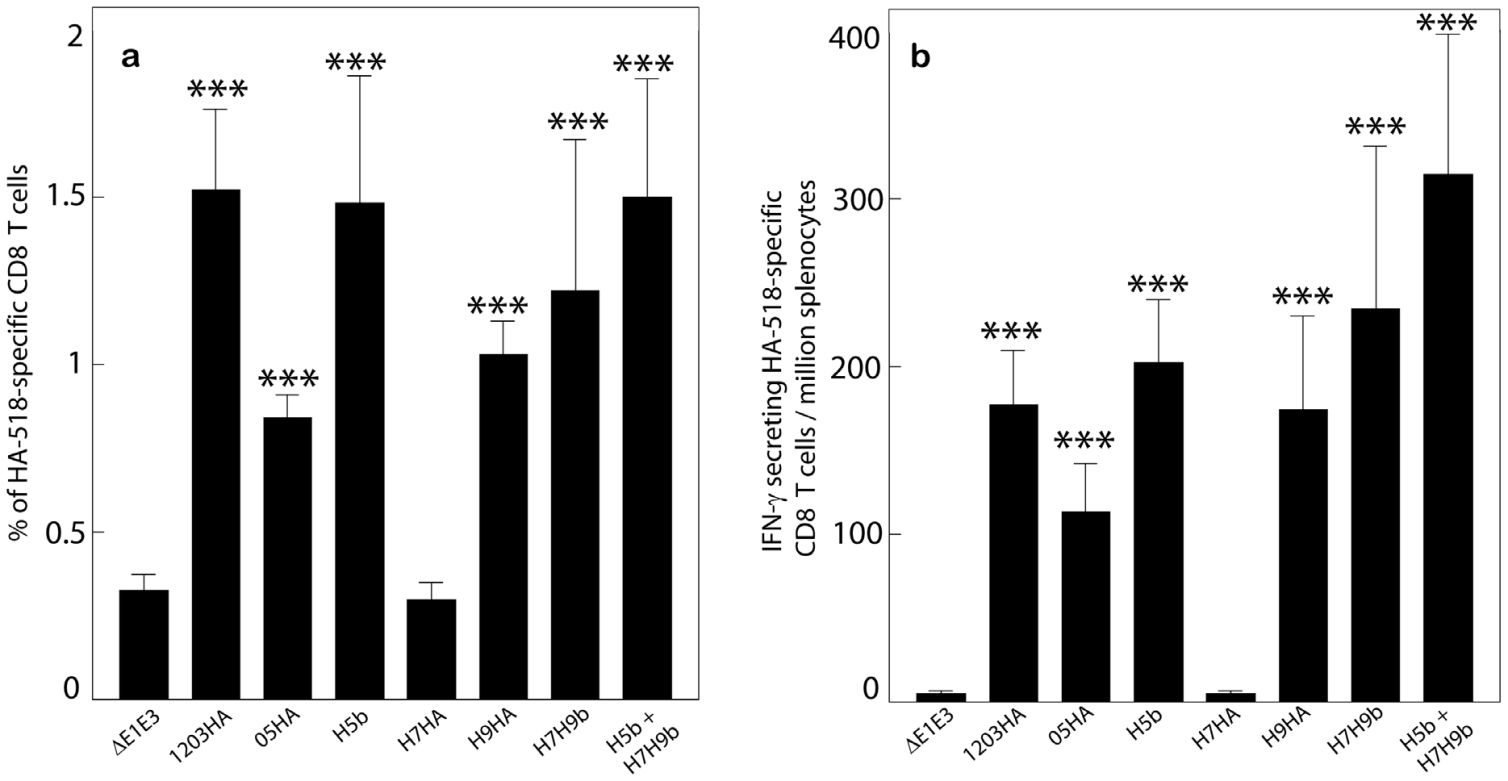
Induction of HA518 epitope pentamer-specific CD8+ T cells and ELISpot measurements of IFN-γ expression in spleen cells of vaccinated mice. Mice (7 animals/group) were immunized with 1203HA, 05HA, H5b, H7HA, H9HA, H7H9b, H5b+H7H9b or ΔE1E3 twice at four-week intervals. At four weeks after booster inoculation, the animals were euthanized, and the spleens were collected for the evaluation of HA-specific CMI responses using HA518 epitope-specific pentamer staining (a) and IFN-γ-ELISpot assay (b) as described under Materials and Methods. The data represent mean± standard deviation (SD) from 7 animals/group. ***; *P*≤0.005. 1203HA, HAd-1203HA; 05HA, HAd-05HA; H5b, HAd-1203HA-05HA; H7HA, HAd-H7HA; H9HA, HAd-H9HA; H7H9b, HAd-H7HA-H9HA; H5b+H7H9b; HAd-1203HA-05HA+HAd-H7HA-H9HA; ΔE1E3, HAd-ΔE1E3.

Cell-mediated immune (CMI) responses induced against influenza has been shown to aid in the clearance of influenza virus infection [28]. To examine the induction of CMI responses against influenza in immunized mice, the spleens were collected four weeks following the second immunization and analyzed for an HA-specific CMI response using anti-influenza CD8 pentamer staining for the HA518 epitope conserved between H5 and H9, but not H7 subtypes, and interferon-gamma (IFN-γ)-ELISpot assays. Consistent with earlier findings [19] vaccination with a monovalent construct (1203HA, 05HA, or H9HA) induced substantial levels of HA518-specific CD8 T cells compared with the empty vector control group (ΔE1E3) [Fig. 2a]. As expected, H7HA vaccine did not induce a HA518 epitope-specific CMI response. No significant differences in the number of HA518 epitope-specific CD8+ T cells were observed between the monovalent and multivalent HA vaccine groups. Furthermore, increased numbers of interferon-γ-secreting cells were detected in response to the HA518 epitope peptide in the spleen cells of mice vaccinated with AdV-based monovalent and bivalent vaccines compared with those inoculated with ΔE1E3 (*P*<0.005) [Fig. 2b].

### Protection Conferred by AdV Vectors Expressing HA of H5, H7 and/or H9 Avian Influenza Viruses Following Challenge with Antigenically Distinct Influenza Viruses

**Figure 3:**
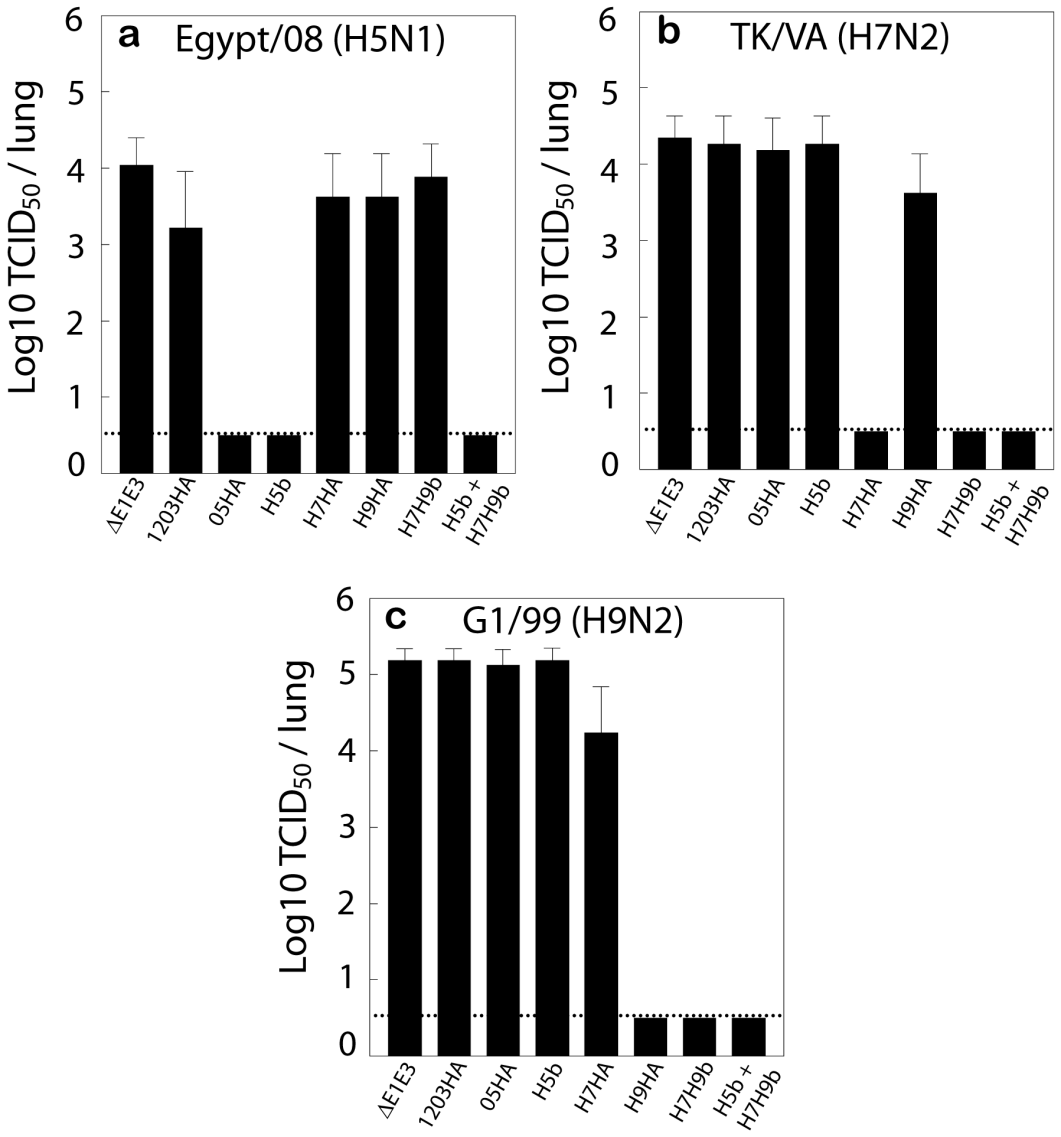
Protective efficacy of AdV vector-based vaccines against challenge with influenza viruses from H5, H7, and H9 subtypes. Mouse groups were immunized with 1203HA, 05HA, H5b, H7HA, H9HA, H7H9b, H5b+H7H9b or ΔE1E3 twice at four-week intervals. Four weeks post-booster inoculation, the mice (5 animals/group) were challenged with one of the following viruses: Egypt/08 (a), TK/VA (b), or G1/99 (c). Three days after challenge, the animals were euthanized, and the lung virus titers were determined as described under Materials and Methods. The data represent the mean virus titers ± standard deviation (SD). The detection limit of the lung viral titer was 0.5 Log_10_ TCID_50_/ml. 1203HA, HAd-1203HA; 05HA, HAd-05HA; H5b, HAd-1203HA-05HA; H7HA, HAd-H7HA; H9HA, HAd-H9HA; H7H9b, HAd-H7HA-H9HA; H5b+H7H9b; HAd-1203HA-05HA+HAd-H7HA-H9HA; ΔE1E3, HAd-ΔE1E3; Egypt/08, A/Egypt/3300-NAMRU3/2008 (H5N1)-PR8-IDCDC-RG13; TK/VA, A/turkey/Virginia/2002 (H7N2)-PR8-IBCDC-5; G1/99, A/Hong Kong/1073/1999 (H9N2).

To determine the efficacy of AdV vector-based vaccines to confer protection against influenza virus challenge animals immunized with 1203 HA, 05HA, H7HA, H9HA, H5b, H7H9b, or H5b+H7H9b were challenged i.n. with 100 MID_50_ of Egypt/08, TK/VA, or G1/99 influenza virus. The protection efficacy was determined by assessing virus titers in the lungs collected three days after challenge. Mean lung virus titers of at least 4 log_10_ TCID_50_/ml were detected in mice that received ΔE1E3 and were challenged with any of the three viruses. In contrast, mice immunized with monovalent vaccines (05HA, H7HA, or H9HA) had no detectable virus in the lungs following challenge with a heterologous virus from the same subtype, but no cross-protection was observed in mouse groups challenged with a heterologous influenza subtype (Fig. 3). Furthermore, the group vaccinated with a bivalent vaccine (H5b) had lung virus titers below the detection limit when challenged with the Egypt/08 virus (Fig. 3a), but the titers remained similar to ΔE1E3 group when challenged with the TK/VA or G1/99 influenza virus (Fig. 3b and 3c). Similarly, animals vaccinated with a bivalent vaccine (H7H9b) had lung virus titers below the detection limit when challenged with the TK/VA or G1/99 influenza virus (Fig. 3b and 3c), but the titers remained similar to ΔE1E3 group when challenged with the Egypt/08 virus (Fig. 3a). Mice vaccinated with the combination vaccine (H5b+H7H9b) had no detectable virus in the lung samples following challenge with any of the three avian influenza virus subtypes, Egypt/08, TK/VA, and G1/99 (Fig. 3a, 3b and 3c).

### Induction of Antibodies Against the HA Stalk Region Following Immunization with AdV Vectors Expressing HA of H5, H7 and/or H9 Avian Influenza Viruses

**Figure 4:**
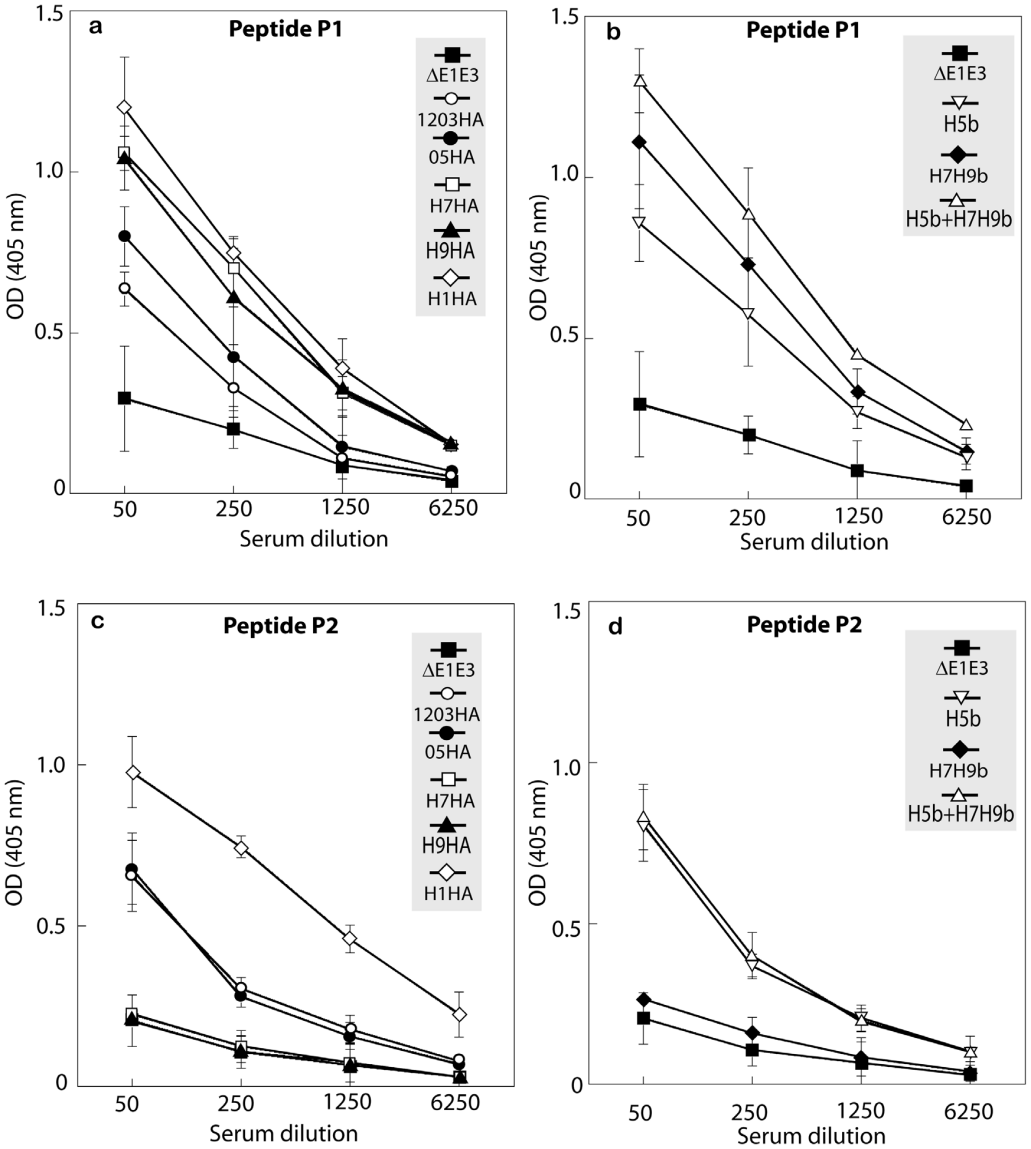
Induction of antibodies against the HA stalk region following immunization with AdV vector-based vaccines. Mice (7 animals/group) were immunized with 1203HA, 05HA, H5b, H7HA, H9HA, H7H9b, H1HA, H5b+H7H9b, or ΔE1E3 twice at four-week intervals. Four weeks post-booster inoculation, serum samples were obtained for determining ELISA antibody titers against peptides P1 (a and b) and P2 (c and d) as described under Materials and Methods. The data represent mean titers ± standard deviation (SD) from 7 animals per group. 1203HA, HAd-1203HA; 05HA, HAd-05HA; H5b, HAd-1203HA-05HA; H7HA, HAd-H7HA; H9HA, HAd-H9HA; H7H9b, HAd-H7HA-H9HA; H1HA, HAd-H1HA; H5b+H7H9b; HAd-1203HA-05HA+HAd-H7HA-H9HA; ΔE1E3, HAd-ΔE1E3.

Antibodies with the potential to neutralize a broad spectrum of influenza A viruses have been shown to target the conserved stalk region of HA [29]–[32]. The epitopes recognized by two broadly cross-protective monoclonal antibodies, 12D1 and CR6261, were shown to be present in the long alpha-helix (LAH) region in the HA stalk. The monoclonal antibody 12D1 binds to a linear epitope present between amino acids 76–106 in the stem of HA, while another monoclonal antibody CR6261 binds to two regions - a linear epitope in the A-helix and an adjacent confirmation epitope in the HA1 region. To determine whether immunization with AdV vector/s expressing HA will induce antibodies against the conserved stalk region of HA, sera from vaccinated mice were tested by ELISA using synthetic peptides, P1 and P2, containing the neutralizing epitopes recognized by the 12D1 or CR6261 monoclonal antibody, respectively. Since a recent study [30] demonstrated high levels of HA stalk-specific antibodies in persons infected with a 2009 pandemic H1N1 virus, serum from mice immunized with a H1HA vaccine (AdV vector-based vaccine against the 2009 H1N1 pandemic virus) was also tested for reactivity against the P1 or P2 peptides. Immunization of mice with any of the monovalent vaccine constructs (1203HA, 05HA, or H1HA) induced high levels of antibodies to both P1 and P2 peptides with the highest reactivity observed with H1HA (Fig. 4a and 4b). Vaccination with H7HA and H9HA induced antibodies against the P1 peptide only. Furthermore, vaccination with the bivalent HA vaccines (H5b or H7H9b) further enhanced the levels of P1 and/or P2 peptide-specific antibodies compared to the groups receiving a monovalent vaccine and the highest levels were observed with the multivalent vaccine [H5b+H7H9b] (Fig. 4c and 4d).

### Further Broadening the Vaccine Coverage by Inclusion of H1HA with the Multivalent Vaccine (H5b+H7H9b)

Since the H1HA vaccine induced significantly high level of antibodies directed against the stalk region of HA, the impact of H1HA in a multivalent vaccine formulation was evaluated. Mice immunized either with a H1HA vaccine or a combination of AdV vectors (H5b+H7H9b+H1HA) developed substantial levels of virus neutralization antibodies against pH1N1 in H1HA immunized animals or pH1N1, Egypt/08, TK/VA, or G1/99 in H5b+H7H9b+H1HA immunized animals (Table 2). Furthermore, animals immunized with H1HA or H5b+H7H9b+H1HA were completely protected against challenge with wild type pH1N1virus. However, no cross-protection was observed when challenged with the H3N2 reassortant virus, X-31.

**Table 2:** Virus neutralization antibody titers and virus lung titers in mice vaccinated with AdV vector-based vaccines and challenged with influenza viruses from H1, H5, H7, H9, and H3 subtypes.

Vaccine Group	pH1N1		Egypt/08 (H5N1)		TK/VA (H7N2)		G1/99 (H9N2)		X-31 (H3N2)	
	VN	VLT	VN	VLT	VN	VLT	VN	VLT	VN	VLT
**ΔE1E3**	≤10	5.2±0.08	≤10	4.2±0.40	≤10	4.5±0.29	≤10	5.2±0.16	≤10	5.6±0.54
**H1HA**	1280	<0.50	≤10	3.62±0.56	≤10	3.72±0.30	≤10	4.54±0.42	≤10	5.6±0.54
**H5b+H7H9b** **+H1HA**	960	<0.50	50	<0.50	113	<0.50	40	<0.50	≤10	5.2±0.10

Mouse groups were immunized with H1HA, H5b+H7H9b+H1HA, or ΔE1E3 twice at four week intervals. Four weeks post-booster inoculation, serum samples were obtained from all animals for determining neutralizing antibody titers (VN) by virus microneutralization assay. Four weeks after the last immunization, mice (5 animals/group) were challenged with one of the following viruses: Egypt/08, TK/VA, G1/99, pH1N1, or X-31. Three days after challenge, the animals were euthanized, and the virus lung titers (VLT) were determined as described under Materials and Methods**.** Antibody titers are shown as geometric mean values and the virus titers are shown as a mean Log_10_ TCID_50_ **±** standard deviation **(**SD). The detection limit of the lung viral titer was 0.5 Log_10_ TCID_50_/ml. H1HA, HAd-H1HA; H5b+H7H9b+H1HA, HAd-1203HA-05HA+HAd-H7HA-H9HA+HAd-H1HA; ΔE1E3, HAd-ΔE1E3; pH1N1, A/California/08/2009 (H1N1); Egypt/08, A/Egypt/3300-NAMRU3/2008 (H5N1)-PR8-IDCDC-RG13; TK/VA, A/turkey/Virginia/2002 (H7N2)-PR8-IBCDC-5; G1/99, A/Hong Kong/1073/1999 (H9N2); X-31, A/Aichi/2/1968 (H3N2)-PR8.

### Inclusion of NP Enhances Vaccine Efficacy Against Heterosubtypic Virus Challenge

**Figure 5:**
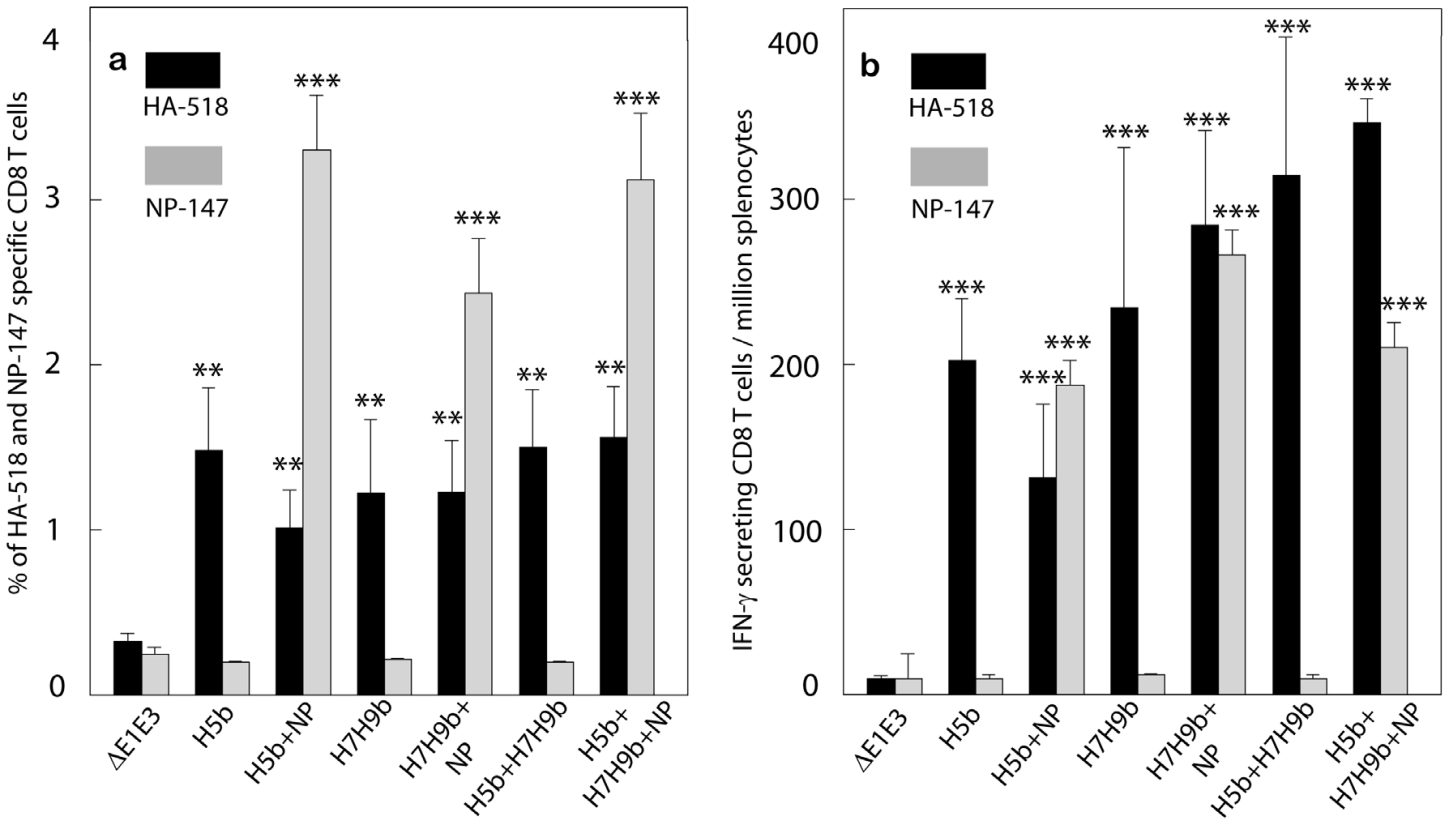
HA518 and NP147 epitope-specific CD8+ T cells and ELISpot measurements of IFN-γ expression in spleen cells of vaccinated mice. Mice (7 animals/group) were immunized with H5b, H7H9b, H5b+H7H9b, H5b+NP, H7H9b+NP, H5b+H7H9b+NP or ΔE1E3 twice at four-week intervals. At four weeks after booster inoculation, the animals were euthanized, and the spleens were collected for the evaluation of HA-specific and NP-specific CMI responses using pentamer staining (a) and IFN-γ-ELISpot assay (b) as described under Materials and Methods. The data represent mean± standard deviation (SD) from 7 animals/group. **; *P*<0.010 and ***; *P*≤0.005 compared to ΔE1E3. H5b, HAd-1203HA-05HA; H7H9b, HAd-H7HA-H9HA; H5b+H7H9b; HAd-1203HA-05HA+HAd-H7HA-H9HA; H5b+NP, HAd-1203HA-05HA+HAd-NP; H7H9b+NP, HAd-H7HA-H9HA+HAd-NP; H5b+H7H9b+NP, HAd-1203HA-05HA+HAd-H7HA-H9HA+HAd-NP; ΔE1E3, HAd-ΔE1E3.

NP is a multifunctional internal protein which encapsulates the viral RNA and plays an important role in the influenza virus life cycle [33] and is conserved across avian and human influenza A subtypes, thus making it a good target for the induction of a broadly protective immunity against influenza A viruses. However, on its own, NP provides incomplete protection [34], [35]. In a previous study from our lab [19], we demonstrated that animals immunized with only an AdV vector expressing NP resulted in an approximately 1.6–3.5-log reduction in the lung virus titers following challenge. Due to the results obtained from this previous study and in order to reduce the number of animals needed for the experiment, we saw no need to include a NP alone vaccine group in our studies. To assess the potential of NP in inducing heterosubtypic immunity, mice were immunized with bivalent or multivalent HA vaccine alone or in combination with the NP vaccine (H5b, H7H9b, H5b+ H7H9b, H5b+NP, H7H9b+NP, or H5b+ H7H9b +NP). Blood samples were collected for the evaluation of humoral immune responses, and the spleens were collected for the evaluation of CMI responses. In general, there was a slight decrease in virus-neutralization and HI titers in the presence of NP, but these changes were not statistically significant (Table 3). Both HA518 (*P*≤0.010) and NP147 (*P*≤0.005) epitope-specific CD8+T cells were observed in all the vaccine groups but not in the groups that received ΔE1E3 (Fig. 5a). Consistent with the humoral response data, inclusion of NP did not have a negative effect on the induction of HA-specific CD8 T cell responses in any of the vaccine groups. Furthermore, significantly (*P*≤0.005) increased numbers of both HA518-specific and NP147-specific interferon-γ-secreting cells were detected in spleen cells of all vaccine groups compared to the group receiving only ΔE1E3 (Fig. 5b). Although the levels of NP-specific CD8 T cells were higher compared to the level of HA-epitope-specific CD8 T cells, there were no significant differences in the frequency of antigen-specific CD8 T cells among the vaccine groups.

**Table 3:** Virus neutralization and hemagglutination inhibition antibody titers in mice vaccinated with AdV vector-based vaccines.

Vaccine group	VN/1203 (H5N1)		Egypt/08 (H5N1)		TK/VA (H7N2)		G9/97 (H9N2)		G1/99 (H9N2)		pH1N1		X-31 (H3N2)	
	HI	VN	HI	VN	HI	VN	HI	VN	HI	VN	HI	VN	HI	VN
**ΔE1E3**	≤10	≤10	≤10	≤10	≤10	≤10	≤10	≤10	≤10	≤10	≤10	≤10	≤10	≤10
**H5b**	194	302	67	107	≤10	≤10	≤10	≤10	≤10	≤10	≤10	≤10	≤10	≤10
**H5b+NP**	96	290	40	86	≤10	≤10	≤10	≤10	≤10	≤10	≤10	≤10	≤10	≤10
**H7H9b**	≤10	≤10	≤10	≤10	262	422	951	1529	48	99	≤10	≤10	≤10	≤10
**H7H9b+NP**	≤10	≤10	≤10	≤10	215	289	780	1280	71	89	≤10	≤10	≤10	≤10
**H5b+H7H9b**	148	211	44	52	144	160	861	905	52	97	≤10	≤10	≤10	≤10
**H5b+H7H9b+NP**	112	160	42	40	121	140	485	538	40	50	≤10	≤10	≤10	≤10
**H5b+H7H9b** **+H1HA**	194	137	50	40	113	105	844	718	40	52	640	960	≤10	≤10
**H5b+H7H9b** **+H1HA+NP**	130	80	35	30	80	69	269	320	32	32	400	480	≤10	≤10

Mice (7 animals/group) were immunized with H5b, H7H9b, H5b+H7H9, H5b+H7H9b+H1HA, H5b+NP, H7H9b+NP, H5b+H7H9b+NP, H5b+H7H9b+H1HA+ NP, or ΔE1E3 twice at 4 week intervals. Four weeks post-booster inoculation, serum samples were obtained for determining virus neutralizing (VN) and hemagglutination inhibition (HI) antibody titers. The data are shown as the geometric mean titers. H5b, HAd-1203HA-05HA; H7H9b, HAd-H7HA-H9HA; H5b+H7H9b; HAd-1203HA-05HA+HAd-H7HA-H9HA; H5b+H7H9b+H1HA, HAd-1203HA-05HA+HAd-H7HA-H9HA+HAd-H1HA; H5b+NP, HAd-1203HA-05HA+HAd-NP; H7H9b+NP, HAd-H7HA-H9HA+HAd-NP; H5b+H7H9b+NP; HAd-1203HA-05HA+HAd-H7HA-H9HA+HAd-NP; H5b+H7H9b+H1HA+NP; HAd-1203HA-05HA+HAd-H7HA-H9HA+HAd-H1HA+HAd-NP; ΔE1E3, HAd-ΔE1E3; VN/1203, A/Vietnam/1203/2004 (H5N1)-PR8/CDC-RG; Egypt/08, A/Egypt/3300-NAMRU3/2008 (H5N1)-PR8-IDCDC-RG13; TK/VA, A/turkey/Virginia/2002 (H7N2)-PR8-IBCDC-5; G9/97, A/chicken/Hong Kong/G9/1997 (H9N2)-PR8-IBCDC-2; G1/99, A/Hong Kong/1073/1999 (H9N2); pH1N1, A/California/08/2009 (H1N1); X-31, A/Aichi/2/1968 (H3N2)-PR8.

To determine whether the addition of NP to the multivalent vaccines provided cross-subtype protection, immunized mouse groups were challenged with 100 MID_50_ of pH1N1, X-31, Egypt/08, TK/VA, or G1/99 viruses. As expected, the lung virus titers were below the detection limit in mice immunized with a vaccines containing or not containing NP following challenge with a virus from the same subtype as the vaccine. However, when challenged with a virus from a different subtype than the vaccine formulation, the virus titers were significantly reduced only in the vaccine groups supplemented with the NP vaccine (Table 4). Vaccination with H5b+NP reduced the replication of TK/VA, G1/99, pH1N1, or X-31 in the lungs by approximately 2.6, 2.4, 0.6, or 1.2 logs, respectively, compared to vaccination with the H5b vaccine. Similarly H7H9b+NP vaccination reduced the replication of VN/1203, pH1N1, or X-31 by 1.7, 2.4, or 1.9 logs, respectively, compared to the H7H9b vaccine. Furthermore, mice vaccinated with H5b+H7H9b+NP showed 2.4 and 1.8 logs reduction in virus titers compared to those receiving H5b+H7H9b following challenge with pH1N1 and X-31, respectively. Overall, these results highlighted the importance of including NP along with HA-containing vaccines to induce heterosubtypic immunity.

**Table 4:** Virus lung titers in vaccinated mice challenged with influenza viruses from H5, H7, H9, H1 and H3 subtypes.

Vaccine group	Egypt/08 (H5N1)	TK/VA (H7N2)	G1/99 (H9N2)	pH1N1	X-31 (H3N2)
**ΔE1E3**	4.2±0.40	4.5±0.29	5.2±0.16	5.2±0.08	5.6±0.54
**H5b**	<0.50	4.5±0.37	5.0±0.16	5.3±0.10	5.6±0.50
**H5b+NP**	<0.50	2.44±0.48**	2.54±0.42**	4.6±0.50	4.34±0.08
**H7H9b**	4.2±0.40	<0.50	<0.50	5.2±0.10	5.32±0.10
**H7H9b+NP**	2.44±0.53*	<0.50	<0.50	2.8±0.50**	3.4±0.50**
**H5b+H7H9b**	<0.50	<0.50	<0.50	5.2±0.10	5.2±0.40
**H5b+H7H9b+NP**	<0.50	<0.50	<0.50	2.6±0.50**	3.4±0.50**
**H5b+H7H9b+H1HA**	<0.50	<0.50	<0.50	<0.50	5.2±0.10
**H5b+H7H9b+H1HA+ NP**	<0.50	<0.50	<0.50	<0.50	3.8±0.89**

Mouse groups were immunized with H5b, H7H9b, H5b+H7H9, H5b+H7H9b+H1HA, H5b+NP, H7H9b+NP, H5b+H7H9b+NP, H5b+H7H9b+H1HA+ NP, or ΔE1E3 twice at 4 week intervals. Four weeks post-booster inoculation, the mice (5 animals/group) were challenged with one of the following viruses: Egypt/08, TK/VA, G1/99, pH1N1, or X-31. Three days after challenge, the animals were euthanized, and the lung virus titers were determined as described under Materials and Methods. The data are shown as the mean Log_10_ TCID_50_ titers ± SD. The detection limit of the lung viral titer was <0.5 Log_10_ TCID_50_/ml (indicated as <0.50).

* , *P*≤0.050 and **, *P*<0.010 compared to ΔE1E3 control. H5b, HAd-1203HA-05HA; H7H9b, HAd-H7HA-H9HA; H5b+H7H9b; HAd-1203HA-05HA+HAd-H7HA-H9HA; H5b+H7H9b+H1HA, HAd-1203HA-05HA+HAd-H7HA-H9HA+HAd-H1HA; H5b+NP, HAd-1203HA-05HA+HAd-NP; H7H9b+NP, HAd-H7HA-H9HA+HAd-NP; H5b+H7H9b+NP; HAd-1203HA-05HA+HAd-H7HA-H9HA+HAd-NP; H5b+H7H9b+H1HA+NP; HAd-1203HA-05HA+HAd-H7HA-H9HA+HAd-H1HA+HAd-NP; ΔE1E3, HAd-ΔE1E3; Egypt/08, A/Egypt/3300-NAMRU3/2008 (H5N1)-PR8-IDCDC-RG13; TK/VA, A/turkey/Virginia/2002 (H7N2)-PR8-IBCDC-5; G1/99, A/Hong Kong/1073/1999 (H9N2); pH1N1, A/California/08/2009 (H1N1); X-31, A/Aichi/2/1968 (H3N2)-PR8.

## Discussion

One of the vaccine strategies during an influenza pandemic scenario would be to immunize the human population with a vaccine which offers some level of cross-protection and could prime for subsequent responses to a strain-matched vaccine when it becomes available. Importance of prior exposure to antigenically related influenza viruses (either by vaccination or natural infection) in mediating protection against pandemic virus infection was clearly demonstrated during the 2009 pH1N1pandemic [36], [37]. Furthermore, priming with an antigenic variant H5N1 virus vaccine (clade 0) was found to enhance antibody responses induced following single dose vaccination with a H5N1 (clade 1) virus vaccine in humans [38], [39].

Earlier we had demonstrated the feasibility of the AdV vector-based vaccine approach for pandemic preparedness against H5N1 influenza viruses [19], [21], [27], [40]. Here, we extend this approach to cover a wide range of potential pandemic influenza viruses originating from the avian reservoir which include genetic drift variants of H5 subtype and potential pandemic strains from H7 or H9 subtypes. Monovalent, bivalent, or multivalent AdV vector-based vaccines expressing HA of H5, H7, or/and H9 subtypes of avian influenza were generated and evaluated for their immunogenicity and protective efficacy against a homologous or heterologous subtype. All AdV vector-based HA vaccines (monovalent, bivalent, or multivalent) induced HA-specific humoral and cellular immune responses and conferred protection against challenge with an influenza virus subtype provided the same HA subtype was included in the vaccine formulation. Inclusion of more than one HA in the vaccine formulation did not significantly impact the development of immune responses against other HA components, but increased the breadth of protective efficacy. To further enhance the cross-protective immunity and effectiveness of the AdV vector-based HA vaccines, we also included the conserved internal protein, NP, in the vaccine formulation. Inclusion of NP in the HA-based vaccines broadened the spectrum of cross-protection against heterosubtypic virus challenge.

Consistent with previous findings [19], vaccination with a monovalent H5 vaccine induced humoral immune responses against a homologous influenza virus, but no cross-reactivity was observed against heterologous influenza subtypes. Despite the lack of humoral responses, mice vaccinated with 05HA had no detectable virus in the lungs after challenge with an antigenically distinct Egypt/08 virus, indicating that immune effectors other than the virus-neutralizing antibody response may have contributed to the viral clearance. However, mice vaccinated with the 1203HA vaccine were not protected against the challenge with Egypt/08. In comparison, the bivalent H5 vaccine (H5b) induced humoral immune responses against both homologous and heterologous influenza viruses of the same subtype and provided complete protection against challenge with Egypt/08 virus. Mice vaccinated with a H7+H9 vaccine (H7H9b) elicited antibodies against both homologous and heterologous influenza viruses of H7 and H9 subtypes and were protected against a heterologous virus challenge.

Although antibody titers against the homologous subtype induced by monovalent vaccines were higher, the bivalent vaccine further broadened vaccine efficacy by inducing humoral responses against both homologous and antigenically distinct virus strain of the same subtype. Furthermore, when used together as a multivalent vaccine composition, protective immune responses against all the three avian influenza subtypes were induced. Although, the antibody titers (both virus neutralization and HI titers) in the combination vaccine group were two-fold lower compared to the bivalent vaccines administered separately, the difference was not statistically significant (P>0.05). Further inclusion of an AdV vector-based H1N1 vaccine into the multivalent vaccine composition resulted in an even broader multi-subtype vaccine that also elicited protective responses against the 2009 pandemic H1N1 virus.

Unlike the variable head region of HA, the stalk region is relatively conserved across influenza A viruses. Several recent studies have demonstrated the importance of HA stalk-specific antibodies in providing heterosubtypic protection. [24], [30], [41]–[44]. Furthermore, it is widely believed that vaccine approaches capable of inducing antibodies directed at the HA stalk region could provide protection against a wide range of emerging influenza A viruses. In our study, we observed high levels of antibodies directed against the HA stalk region. Vaccination with AdV expressing HAs from different influenza virus subtypes seemed to enhance the level of anti-HA stalk antibodies. Interestingly, H1HA vaccine induced the highest level of HA stalk-specific antibodies. These results are consistent with those of Pica et al [30] who observed high levels of antibodies against the long α-helix (LAH) region of HA stem in patients-infected with pH1N1. Recently a peptide-based vaccine targeting the LAH showed broad protection against multiple influenza viruses [24]. In our study we did not observe heterosubtypic protection due to the HA stalk-specific antibodies. The discrepancies may be because of a higher dose of challenge virus in our study and the differences in the protective endpoints measured (e.g., lung virus titers rather than protection from death) compared to the published studies.

The cellular immune responses induced against NP play an important role in the virus clearance from the lungs, and, therefore, are critical for recovery from influenza virus infections. Since NP is highly conserved across influenza A virus subtypes, the CMI responses generated against it are usually broadly cross-protective [13], [19]. It is widely believed that such cross-reactive NP-specific CMI responses have tremendous potential to reduce the impact of an influenza pandemic [45], [46]. In this study, inclusion of NP along with bivalent or multivalent-HA vaccines did enhance vaccine efficacy by inducing partial cross-protection against challenge with influenza A viruses from a different subtype. The role of NP in inducing heterosubtypic immunity has been previously demonstrated [47]. Although the precise mechanism for the NP-induced heterosubtypic immunity is not yet clear, it is thought to be mediated by -NP-specific cellular and humoral immune responses [48]. Indeed, the mice groups that received an NP vaccine had substantial levels of NP147 epitope-specific CD8 T cells which secreted IFN-γ.

Due to high prevalence of AdV infections in humans, there is variable levels of pre-existing AdV immunity in the majority of human population and it is believed that it can adversely impact the vaccine efficacy of AdV vector-based vaccines [49]–[51].Earlier we have demonstrated in a mouse model that AdV vector immunity at the level of approximately 1500 virus-neutralization titer can be overcome by either change in the route of vaccine administration or increasing the vector dose [40]. The implication of vector immunity in inhibiting AdV vector-based gene delivery in humans is not well understood. The failure of Merck’s HIV-1 STEP trial [52], [53]was not due to AdV vector immunity as initially thought. Furthermore, results from other HIV-1 vaccine clinical trials have indicated no association between pre-existing AdV seropositivity and HIV-I acquisition [54], [55]. In addition, a Phase I study involving twenty-four healthy adult volunteers indicated that there was no correlation between neutralizing antibody titers against AdV and the immune response to the vaccine antigen [56]. Various strategies to overcome vector immunity are described elsewhere [57].

Eventually the HA and/or NP gene cassettes of H5, H7, and H9 avian influenza subtypes will be expressed in a single AdV vector to reduce the vaccine dose and avoid the complexity of vaccine production, testing and formulation. It has been demonstrated that an AdV-based influenza vaccine dose of 5×10^8^ VP when given i.n. resulted in approximately 80% seroconversion (at least four-fold rise in HI titers) in humans without any major safety concerns [56]. In another study, two doses of 3×10^10^ VP of an AdV vector expressing the *M. tuberculosis* antigens Ag85A, Ag85B, and TB10.4 resulted in a more than 50-fold increase in the frequencies of TB-specific CD8+ T cells in humans primed with the BCG vaccine [58]. Since the potential of AdV vector-based therapies are currently being evaluated in a number of clinical trials, the biopharmaceutical industry has the technology for the large scale purification of AdV vectors. More than 1×10^18^ purified VP are projected to be produced from a single 10,000 L bioreactor within 7–8 weeks [50]. This yield should be equivalent to ten million vaccine doses if 1×10^11^ VP are used per individual as a one- or two-dose regiment suggesting that AdV vector-based vaccines can be produced at large scale for meeting the pandemic influenza vaccine requirement.

In summary, our findings demonstrate the feasibility of a multivalent vaccine approach for pandemic preparedness against multiple avian influenza virus subtypes. Such a multicomponent vaccine could provide a first line of defense following the emergence of a pandemic threat and/or could be used to prime the population in the inter-pandemic period to induce some level of immunity that would be boosted following infection with the pandemic strain.
